# Racial and Ethnic Differences in Risk Factors and Prodromal Symptoms That Predict Eating Disorder Onset: A 3-Year Prospective Study of Adolescent Girls and Young Women

**DOI:** 10.3390/jcm15103872

**Published:** 2026-05-18

**Authors:** Yuko Yamamiya, Eric Stice

**Affiliations:** 1 Division of Liberal Arts (Psychology), Temple University Japan Campus, Tokyo 154-0004, Japan; yuko.yamamiya@tuj.temple.edu; 2 Department of Psychiatry and Behavioral Sciences, Stanford University, Stanford, CA 94305, USA

**Keywords:** anorexia nervosa, bulimia nervosa, binge eating disorder, purging disorder, risk factors, prodromal symptoms, women, ethnicity/race, longitudinal study

## Abstract

**Background/Objective:** Our current knowledge regarding ethnic/racial differences in the incidence of and risk factors for eating disorder onset is relatively limited. We examined whether the baseline prevalence and incidence of onset of any eating disorder over follow-up and the risk factors that predict future onset of any eating disorder differ for various ethnic/racial groups. **Methods:** Data were collected from females across a wide age range (*N* = 1952; White = 61%, Hispanic = 17%, Asian = 14%, Black = 5%, and Native American = 3%; *M* baseline age = 19.7, *SD* = 5.7; baseline age range: 13–64) who completed self-report questionnaires and a diagnostic interview at baseline and then annually over 3 years. We ran two chi-square tests that examined how ethnicity/race were related to eating disorders at baseline and future onset as well as a series of logistic regression models that tested whether baseline risk factors and prodromal symptoms were differentially related to future eating disorder onset across ethnic/racial groups. **Results:** The diagnostic prevalences as well as the predictive relationship of a risk factor and a prodromal symptom with eating disorder onset were very similar across ethnic/racial groups, with only one instance where the magnitude of the predictive effects differed across two ethnic/racial groups; lower zBMI was predictive among White women, whereas higher zBMI was predictive among Black women. **Conclusions:** Overall, risk factors and prodromal symptoms are similar across the examined ethnic/racial groups, suggesting that we can implement the same prevention programs for women with the same risk factors, regardless of their ethnic/racial identities.

## 1. Introduction

Anorexia nervosa (AN), bulimia nervosa (BN), binge eating disorder (BED), as well as subthreshold cases of these disorders and purging disorder (PD), classified as Other Specified Feeding and Eating Disorders (OSFEDs) as per DSM-5 [1], are associated with various psychological and medical problems [2,3,4,5]. Individuals with OSFEDs frequently exhibit comparable functional impairment with those with full-fledged eating disorders [1,4,6], and more than 50% of individuals who seek eating disorder treatment are diagnosed with OSFED [7,8], highlighting the importance of elucidating predictors of the full range of eating disorders.

How ethnicity/race predicts eating disorders remains inconclusive. While White women are traditionally thought to be at a higher risk of developing eating disorders compared to other ethnic/racial groups, recent etiological findings have been inconsistent. Some studies have indicated that the prevalence of eating disorders differs across ethnic/racial groups [9,10], whereas others have found no such differences [11,12]. One thing to note is that although the epidemiological studies we have reviewed here reported the prevalence of eating disorders using structured clinical interviews, the instruments were varied, with some based on the DSM-IV criteria whereas others were based on the DSM-5 criteria. Therefore, ethnic/racial differences in eating disorder prevalence and incidence should be examined in a larger prospective study.

Further, risk factors, such as pursuit of the thin ideal, body dissatisfaction, dieting, negative affect, psychosocial impairment, and prodromal symptoms, such as binge eating, compensatory weight control behaviors, overvaluation of weight/shape, and fear for weight gain at baseline, have been found to increase risk for future onset of eating disorders [13,14,15,16]. However, few studies have examined whether baseline risk factors and prodromal symptoms are differentially related to future onset of eating disorders across ethnic/racial groups. If the predictive effects of risk factors or prodromal symptoms differ across ethnic/racial groups, it might be necessary to target distinct risk factors or prodromal symptoms for different ethnic/racial groups.

To address these issues, we collected data from a large community-recruited sample of healthy females across a wide age range in various prevention trials of programs to reduce appearance concerns and examined whether ethnicity/race (1) correlates with baseline prevalence of eating disorders and predicts future onset of eating disorders and (2) moderates the predictive relationship of risk factors and prodromal symptoms with future onset of any eating disorder. Thin-ideal internalization, body dissatisfaction, negative affect, restrictive eating, psychosocial impairment, and body mass index (BMI) were included because they were found to predict future onset of eating disorders in adolescent and young females [15,16,17,18]. Further, all prodromal symptoms of eating disorder required by DSM-5 (i.e., binge eating, compensatory weight control behaviors, overvaluation of weight/shape, and fear for weight gain) were included because research has found that baseline prodromal symptoms predict future onset of eating disorders [15,16]. Based on past findings, we hypothesized that White women show higher baseline prevalence and future onset of eating disorders (incidence). Further, as past studies have indicated that Black and Hispanic women express lower levels of thin-ideal internalization, we hypothesized that the predictive effects of risk factors and prodromal symptoms related to the pursuit of the thin ideal would be weaker for these ethnic/racial groups compared to White women. As nobody has evaluated whether ethnicity/race moderates the effects of the most widely studied risk factors, our analyses should make an important contribution to the literature.

## 2. Materials and Methods

Participants enrolled in one efficacy trial (prior to ClinicalTrials.gov), two effectiveness trials (ClinicalTrials.gov NCT00663754; ClinicalTrials.gov NCT01126918), and one task-shifting implementation trial (ClinicalTrials.gov NCT01949649) were combined for the present study (see [15] for greater details). Our initial sample consisted of 1,952 females across a wide age range (*M* baseline age = 19.7; *SD* = 5.7; baseline age-range: 13–64) with a wide range of ethnic heritage (White = 66%, Asian = 10%, Hispanic = 8%, Black = 5%, Native American = 2%, Pacific Islander = 1%, and Others = 8%). They resided in Texas, Oregon, and Pennsylvania, making the data more representative than if it had been collected from a single region.

Mailings and fliers were sent to high schools and colleges to recruit self-identified females for trials evaluating body acceptance programs. The only inclusion criterion was that participants had to say “yes” to a question if they had body image concerns, which increases risk for eating disorders, making this a high-risk sample. Informed consent was obtained from the participants and from the parents of minor participants.

### 2.1. Measures

#### 2.1.1. Eating Pathology

We used the semi-structured Eating Disorder Diagnostic Interview (EDDI) [17] to assess eating disorder symptoms over the past 3 months at baseline. The same interview was then repeated at each follow-up, covering the time since the previous interview on a month-by-month basis using the timeline follow-back method over 3 years. This data allowed us to determine if participants had threshold or subthreshold eating disorders at baseline or developed any of these disorders over the 3-year period. We did not assess weight history of participants across all trials, making it challenging to diagnose atypical AN. We used DSM-5 diagnostic criteria of threshold and subthreshold eating disorders, as defined in Stice et al. [17]. See Table 1 for the complete diagnostic criteria.

Female assessors who had a B.A./B.S., M.A., or Ph.D. in psychology underwent 24 h of training, during which they learned structured interview skills and diagnostic criteria, observed simulated interviews, and participated in role-playing exercises. They were required to demonstrate inter-rater agreement (κ > 0.80) with supervisors on 12 tape-recorded interviews before they collected data. Annual refresher training was also conducted to prevent diagnostic drift and ensure the reliability and validity of the diagnostic interviews over the follow-up period.

Test–retest reliability was examined using data from 351 participants who repeated the EDDI with the same assessor. One-week test–retest reliability was κ = 0.79 for DSM-5 eating disorder diagnoses. For inter-rater reliability, 330 participants completed the EDDI with a second assessor within one to three days. Inter-rater reliability was κ = 0.75 for DSM-5 eating disorder diagnoses. Furthermore, participants with DSM-5 EDDI-diagnosed eating disorders exhibited greater functional impairment, emotional distress, and service utilization than those without [4].

#### 2.1.2. Thin-Ideal Internalization

Thin-ideal internalization was assessed using the Ideal-Body Stereotype Scale-Revised [17]. This scale consists of six items (e.g., “Slender women are more attractive”) that assess the extent to which a respondent endorses a sociocultural thin ideal, using a 5-point scale (1 = *strongly disagree*, 5 = *strongly agree*). Higher mean scores indicate greater endorsement. The scale has high internal consistency (α = 0.91), 2-week test–retest reliability (*r* = 0.80), and predictive validity for future BN, BED, and PD onset [17]. Cronbach’s α was 0.69 at baseline.

#### 2.1.3. Body Dissatisfaction

Body dissatisfaction was assessed using the Body Dissatisfaction Scale [19], which consists of nine items that assess a respondent’s satisfaction with various body parts (e.g., “How satisfied are you with your: [Weight]”) using a 5-point scale (1 = *extremely dissatisfied*, 5 = *extremely satisfied*). Item scores were reversed prior to analyses, so higher mean scores indicated greater dissatisfaction. The scale has high internal consistency (α = 0.94), 3-week test–retest reliability (*r* = 0.90), and predictive validity for future BN, BED, and PD onset [17]. Cronbach’s α was 0.84 at baseline.

#### 2.1.4. Negative Affect

Different measures were utilized across trials, and their scores were standardized to allow analyses of the combined data. In Trials 1 and 4, negative affect was assessed with 20 relevant items from the Positive Affect and Negative Affect Scale-Revised (PANAS-X) [20], which has shown internal consistency (α = 0.95), 3-week test–retest reliability (*r* = 0.78), and predictive validity for bulimic symptom onset (17). In Trial 2, we used the 20-item Center for Epidemiologic Studies-Depression Scale (CES-D) [21], which has shown internal consistency (α = 0.74–0.91), temporal reliability (2- to 8-week test–retest *r* = 0.51–0.59), and convergent validity with clinician ratings of depressive symptoms (mean *r* = 0.88) [22]. In Trial 3, we used the 21-item Beck Depression Inventory [23], which has shown internal consistency (α = 0.73–0.95), 1-week test–retest reliability (*r* = 0.93), and convergent validity with clinician ratings of depressive symptoms (mean *r* = 0.75) [17,23]. The PANAS-X scores are correlated with depressive symptom measures (mean *r* = 0.63) [20], which enables us to average across the three negative affect assessments. Higher composite scores indicated greater negative affect. This negative affect composite has shown predictive validity for future BN, BED, and PD onset (α = 0.94) [17].

#### 2.1.5. Restrictive Eating

Restrictive eating was assessed using the Dutch Restrained Eating Scale [24]. It consists of nine items (e.g., “I would be happy if I were thin”) that assess how often a respondent engages in dieting behaviors, using a 5-point scale (1 = *strongly disagree*, 5 = *strongly agree*). Higher scores indicate greater restrictive eating. The scale has high internal consistency (α = 0.95), 2-week test–retest reliability (*r* = 0.82), and predictive validity for future BN, BED, and PD onset [17,24]. Cronbach’s α was 0.92 at baseline.

#### 2.1.6. Psychosocial Impairment

We used 17 items from the Social Adjustment Scale-Self Report for Youth to assess psychosocial impairment in the family, peer, romantic, and school domains [25]. This scale has shown internal consistency (α = 0.77), 1-week test–retest reliability (*r* = 0.83), and predictive validity for future AN, BN, BED, and PD onset (α = 0.74) [15,17]. Higher scores indicated greater psychosocial impairment. Cronbach’s α was 0.73 at baseline.

#### 2.1.7. Standardized Body Mass Index (zBMI)

BMI (kg/m^2^) [26] was assessed as a measure of height-adjusted body weight. Portable stadiometers were used to measure height to the nearest millimeter. Moreover, digital scales were used to measure weight to the nearest 0.1 kg. Participants wore light indoor clothing only during the measurements. Measurements of each were taken twice and averaged. BMI has demonstrated convergent validity (*r* = 0.80–0.90) with direct measures of body fat [26] and predictive validity for the future onset of AN [17]. As our sample consisted of females across a wide age range, raw BMI might not be directly comparable across our participants. Therefore, we used zBMI in our analyses. More specifically, for adolescent participants, BMI-for-age z-scores (zBMI) were calculated based on CDC growth charts. For adult participants, BMI was standardized within the sample (z-scores). Participants with missing age data were excluded from analyses involving BMI standardization.

#### 2.1.8. Prodromal Eating Disorder Symptoms

We used baseline EDDI scores to assess prodromal symptoms. Behavioral prodromal symptom was defined as the frequency of binge eating and/or compensatory behavior episodes, including self-induced vomiting, laxative/diuretic abuse, fasting, and excessive exercise. Cognitive prodromal symptom was defined as exhibiting required diagnostic levels of weight/shape overvaluation, fear of weight gain, and/or feeling fat based on the DSM-5 (Table 1). Participants indicated their weight/shape overvaluation on a 7-point scale ranging from 0 = *no importance* to 6 = *supreme importance (nothing is more important in terms of self-evaluation)*. Participants indicated their fear of weight gain on a 7-point scale ranging from 0 = *0 days per week in the past month (no definite fear of fatness or weight gain)* to 6 = *6*–*7 days per week in the past month (definite fear of fatness or weight gain present every day)*. Participants indicated their feeling fat on a 7-point scale ranging from 0 = *0 days per week in the past month (has not felt fat)* to 6 = *6*–*7 days per week in the past month (has felt fat every day)*. We created a prodromal symptom variable by averaging the number of episodes of behavioral symptoms or the severity levels of cognitive symptoms reported at baseline over the past month.

Test–retest reliability was examined using data from 351 participants who repeated the EDDI with the same assessor. One-week test–retest reliability was κ = 0.94 for binge eating, κ = 0.86 for compensatory behavior, κ = 0.80 for overvaluation of weight/shape, κ = 0.89 for fear of weight gain, and κ = 0.83 for feeling fat. For inter-rater reliability, 330 participants completed the EDDI with a second assessor within one to three days. Inter-rater reliability was κ = 0.51 for binge eating, κ = 0.77 for compensatory behavior, κ = 0.89 for overvaluation of weight/shape, κ = 0.86 for fear of weight gain, and κ = 0.85 for feeling fat.

### 2.2. Statistical Methods

#### 2.2.1. Missingness

Missing data occurred for restrictive eating (0.1%), negative affect (0.5%), and the onset of AN (1.5%), BN (1.1%), BED (2.2%), and PD (1.1%). Thus, eating disorder onset variables had the most missing data, but we were reluctant to impute eating disorder onset data based on baseline symptoms. Therefore, we focused on participants with complete data.

#### 2.2.2. Analytical Strategies

First, we examined how ethnicity/race were related to eating disorders at baseline and future onset using a chi-square test. Second, we examined whether baseline risk factors and prodromal symptoms predicting future onset of eating disorder differed across ethnic/racial groups using a series of logistic regressions. As most past studies reported on White women, we considered it important to test whether predictive relations would differ for Asian, Black, Hispanic, and Native American women compared to their White counterparts. We created dummy variables to compare each ethnic/racial group to White participants (i.e., White vs. Asian, White vs. Black, White vs. Hispanic, and White vs. Native American). Additionally, we used mean-centered scores of baseline risk factors and prodromal symptoms to reduce unnecessary collinearity between the main effects and interaction terms. Because we conducted a total of 55 inferential tests, the Benjamini–Hochberg procedure was used to adjust *p*-values in order to control the false discovery rate [27]. More specifically, we ranked *p*-values and adjusted them based on their rank order and the total number of tests, allowing for a controlled proportion of false discoveries (set at FDR = 0.05 by default). Lastly, we reported the odds ratio (OR) as well as success rate difference (SRD) as our effect size, given that our outcome was a dichotomous variable (i.e., the presence or absence of any eating disorder). An SRD of 0.11 indicates a small effect, 0.28 a medium effect, and 0.43 a large effect [28]. The analyses were based on samples with complete data on the variables relevant to each model. As such, sample sizes differed slightly across analyses due to variable-specific missingness. For statistical analyses, we used SPSS version 26 and R version 4.3.0 [29].

## 3. Results

### 3.1. Preliminary Analyses

Participants self-reported their ethnicity/race on a demographic questionnaire. Participants were allowed to report multiple ethnicities/races. For analytic purposes, participants who reported more than one ethnicity/race were assigned to a single ethnic/racial group based on sample distribution to ensure adequate cell sizes for statistical analyses (i.e., were assigned to the smaller ethnic/racial group). Among the 1952 participants, 69 were excluded because they did not specify their ethnicity/race or identified as Pacific Islanders, for which we could not conduct analyses due to the small number of cases. Our final sample consisted of 1883 participants (White = 61%, Hispanic = 17%, Asian = 14%, Black = 5%, and Native American = 3%). This dataset built upon the dataset used in Cheng et al. [11], increasing the sample size from 1177 to 1883. We examined the test with the lowest power in each paper (i.e., the contrast involving White participants vs. the smallest cell size) and averaged the cell sizes, and found that the power to detect a small effect increased from 0.85 to 0.96 [30]. Among the current sample, 328 participants or 17.4% (*M* baseline age = 20.0; SD = 5.06; age range = 13–64) developed any eating disorders over the 3-year follow-up. According to population-level estimates of the lifetime prevalence of all eating disorders (i.e., AN, BN, BED, and OSFED), approximately 8.6% of women develop an eating disorder [31], which is lower than our incidence rate. However, our sample consisted of individuals at risk for eating disorders because body image concerns were an inclusion criterion. Moreover, our incidence rate is similar to the one reported in previous epidemiological studies that recruited community samples [17].

### 3.2. Main Analyses

#### 3.2.1. Baseline Prevalence and Future Onset of Any Eating Disorder Across Ethnic/Racial Groups

We conducted a chi-square test to examine the relationship between ethnic/racial groups and any eating disorders including OSFEDs at baseline due to the differential proportions of eating disorders observed across the groups (Table 2). The results were not significant, with χ^2^(4, *N* = 1883) = 4.45, *p* = 0.348. The same test was repeated to examine the relationship between ethnic/racial groups and the onset of any eating disorder in the follow-up period. The results were again not significant, with χ^2^(4, *N* = 1883) = 9.38, *p* = 0.052.

#### 3.2.2. Predictive Relation of Baseline Risk Factors to Future Onset of Eating Disorders Across Ethnic/Racial Groups

**Figure 1:**
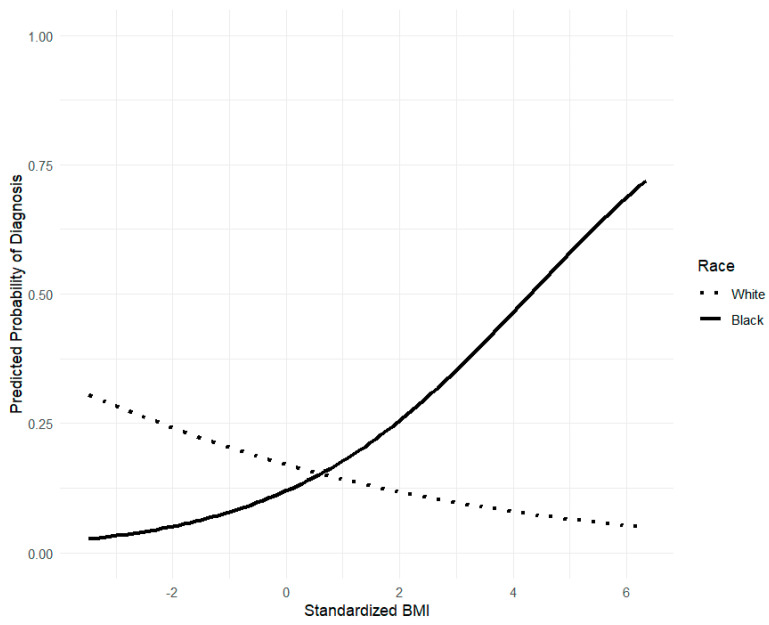
Plot of interactive predictive effect of ethnicity/race and zBMI.

We conducted a series of logistic regression analyses using dummy variables to represent ethnicity/race contrasts and continuous risk factor variables. The main effects of each risk factor for the reference group (i.e., White women) were significant except for zBMI after *p*-values were adjusted. The main effects for White women indicated that the OR for thin-ideal internalization was 2.22 (95% CI [1.58–3.13], adjusted *p* = 0.006) with a small to medium effect (SRD = 0.23). For body dissatisfaction, the OR was 1.95 (95% CI [1.56–2.45], adjusted *p* = 0.006) with a medium effect (SRD = 0.26). For negative affect, the OR was 1.97 (95% CI [1.69–2.30], adjusted *p* = 0.006) with a medium to large effect (SRD = 0.37). For restrictive eating, the OR was 2.37 (95% CI [1.96–2.89], adjusted *p* = 0.006) with a large effect (SRD = 0.40). For psychosocial impairment, the OR was 2.50 (95% CI [1.89–3.32], adjusted *p* = 0.006) with a medium effect (SRD = 0.26). For zBMI, the OR was 0.80 (95% CI [0.68–0.96]), adjusted *p* = 0.039) with a small effect (SRD = 0.12). Thus, restrictive eating and negative affect showed the largest effects based on SRD, whereas psychosocial impairment and restrictive eating showed larger ORs. The mean OR of the main effects for White women was 1.97, whereas the mean SRD was 0.27. On the contrary, all risk factor × ethnicity/race interactive effects were nonsignificant after adjusting *p*-values except for zBMI and ethnicity/race (White vs. Black). The main effect of zBMI for Black women was OR = 1.59 (95% CI [1.01–2.50], *p* = 0.045) with a medium effect (SRD = 0.20). For the interaction, the OR was 1.98 (95% CI [1.22–3.25], adjusted *p* = 0.035), though the effect was rather small (SRD = 0.15). Figure 1 represents the interaction between zBMI and ethnicity/race (White vs. Black) in predicting the future onset of any eating disorders. The mean OR of the interactive effects was 1.13, whereas the mean SRD was 0.28. See Table 3 for the complete results.

#### 3.2.3. Predictive Relation of Baseline Prodromal Symptoms to Future Onset of Eating Disorders Across Ethnic/Racial Groups

We used a similar approach to test whether the predictive effects of prodromal symptoms differed significantly across ethnic/racial groups. The main effects of all prodromal symptoms for the reference group (i.e., White women) were significant after *p*-values were adjusted. The main effects for White women indicated that the OR for binge eating was 1.28 (95% CI [1.20–1.37], adjusted *p* = 0.005) with a medium to large effect (SRD = 0.37). For compensatory behavior, the OR was 1.40 (95% CI [1.27–1.53], adjusted *p* = 0.005) with a medium to large effect (SRD = 0.37). For overvaluation of weight/shape, the OR was 1.72 (95% CI [1.50–1.97], adjusted *p* = 0.005) with a medium to large effect (SRD = 0.39). For fear of weight gain, the OR was 1.32 (95% CI [1.23–1.40], adjusted *p* = 0.005) with a medium effect (SRD = 0.34). For feeling fat, the OR was 1.28 (95% CI [1.19–1.38], adjusted *p* = 0.005) with a medium effect (SRD = 0.30). Thus, both SRD and OR indicated that overvaluation of weight/shape showed the largest effect, followed by compensatory behavior. The mean OR of the main effects for White women was 1.40, whereas the mean SRD was 0.35. On the contrary, all prodromal symptom x ethnicity/race interactive effects were nonsignificant after adjusting *p*-values. The mean OR of the interactive effects was 1.03, whereas the mean SRD was 0.36. See Table 4 for the complete results.

Lastly, we conducted a multivariable logistic regression analysis including all predictors simultaneously, along with zBMI and ethnicity/race, as a post hoc analysis. The results indicated that binge eating (OR = 1.24), compensatory behaviors (OR = 1.09), weight concerns (OR = 1.17), fear of weight gain (OR = 1.08), negative emotionality (OR = 1.23), dietary restraint (OR = 1.41), psychosocial impairment (OR = 1.41), and zBMI (OR = 0.73) remained significant predictors, demonstrating their unique predictive effects above and beyond other variables. In contrast, body fat concerns, thin-ideal internalization, body dissatisfaction, and ethnicity/race were not significant when accounting for shared variance among predictors. Multicollinearity diagnostics indicated no concerns (i.e., all GVIFs < 1.42).

## 4. Discussion

The present study examined whether ethnicity/race correlated with baseline prevalence or predicted future onset of eating disorders and whether risk factors and/or prodromal symptoms differentially predict future onset of any eating disorder across ethnic/racial groups, using White women as the reference group. The results indicated that the baseline prevalence and the future onset of eating disorders are similar across ethnic/racial groups, consistent with other studies [12]. That is, our findings suggest no reliable differences in baseline prevalence and incidence of eating disorders across ethnic/racial groups over a 3-year follow-up, emphasizing the need to refer to other distinguishing factors, not ethnicity/race, to detect high-risk individuals.

Moreover, the predictive effects of prodromal symptoms and risk factors are largely similar across the groups, except for zBMI; the predictive effect of zBMI was different for Black women versus White women. Specifically, a higher baseline zBMI predicts the future onset of eating disorders among Black women, whereas a lower baseline zBMI predicted the future onset of eating disorders among White women. While low body weight may be a prodromal symptom of eating disorders, higher body weight may act as a trigger, as overweight women often face increased pressure to lose weight due to weight-related discrimination and social stigma. Hence, it may be beneficial to incorporate a specific focus on reducing BMI among Black females. As the interaction terms had small effect sizes, we did not miss a substantively meaningful moderation effect due to limited statistical power.

Taken together, prodromal symptoms and risk factors for eating disorders appear to be more similar than different across ethnic/racial groups. Therefore, the same prevention programs may be applied to any young females who exhibit risk factors and/or prodromal symptoms. It should be possible to use prevention programs (e.g., the Body Project) that have reduced future eating disorder onset by 54–77% in controlled trials among various ethnic/racial groups [32]. However, for Black females, implementing dual prevention programs targeting both obesity and eating disorders may be more effective than for other ethnic/racial groups.

There are some limitations of the study that should be noted. First, the number of participants in some ethnic/racial groups was small (e.g., Native American), so the findings may not accurately represent the U.S. population. Furthermore, generalizability is reduced although the use of a high-risk design made it more feasible to address the study aims because of the higher prevalence and incidence of eating disorders among a high-risk sample. Additionally, we needed to examine eating disorders collectively due to the limited sample size. It would be useful if larger studies tested whether the predictive effects of risk factors and prodromal symptoms for each of the four major types of eating disorders differed across ethnic/racial groups. Second, we examined the predictive effects of only six well-known risk factors, so it is possible that the predictive effects of other risk factors may differ across ethnic/racial groups. For instance, a lack of social support and modeling of pathological eating predicted eating disorders among adolescent girls [18]. Moreover, some negative life experiences that are more prevalent among certain ethnic/racial groups (e.g., racial discrimination and food insecurity) may have differential interactive effects. There are also other weight-related and non-weight-related attributes that are highly valued among ethnic/racial minority groups (e.g., skin tone, hair texture, thick bottoms) and that influence the general body image of minority women. Future research should test whether other risk factors interact and show differential predictive effects for ethnic/racial groups. Third, we conducted 55 inferential tests in total, so it is possible that the observed significant findings occurred by chance. To address this, we controlled the false discovery rate using the Benjamini–Hochberg correction.

Despite these limitations, our findings are valuable as this study is novel and unique. To our knowledge, it is the first prospective study to test whether predictive effects of risk factors and prodromal symptoms to future onset of any eating disorder differed across ethnic/racial groups. Additionally, our data were collected from several regions in the U.S., which should increase the generalizability of the findings. Nevertheless, it would be beneficial to replicate the current findings in novel prospective studies.

Regarding clinical implications, the results suggest that implementing the most effective prevention programs may reduce the prevalence of eating disorders in the general population across a range of racial and ethnic groups. However, we found that BMI was predictive of eating disorders in Black women. Obesity was previously regarded as an independent medical issue, but it appears to share common etiologies with eating disorders [33]. However, Black women are often underdiagnosed and underserved for their eating pathologies [34,35], partly because they are known to have lower levels of body dissatisfaction than other ethnic/racial groups, attributed to lower levels of thin-ideal internalization [36,37]. Additionally, ideal body shape is different across ethnic/racial groups; prior research suggests that Black and Hispanic females tend to idealize a thick or shapely body, whereas White and Asian females tend to idealize a thin body [38,39,40]. Hence, it is important to investigate unique predictors across different ethnic/racial groups while addressing all ethnic/racial groups equitably.

## Figures and Tables

**Table 1:** Diagnostic criteria of threshold and subthreshold eating disorders.

Disorder	DSM-5 Criteria
Anorexia nervosa (AN)	BMI less than 85% of what is expected for age, gender, and height. Excessive fear of weight gain/fatness more than 75% of the days for at least three months. Undue influence of body weight and shape on self-evaluation.
Subthreshold AN	BMI between 85% and 90% of what is expected for age, gender, and height. Excessive fear of weight gain/fatness more than 25% of the days for at least three months. Undue influence of body weight and shape on self-evaluation.
Bulimia nervosa (BN)	At least four episodes of uncontrollable binge eating per month for at least three months. At least four episodes of inappropriate compensatory behaviors per month for at least three months. Undue influence of body weight and shape on self-evaluation.
Subthreshold BN	At least two episodes of uncontrollable binge eating per month for at least three months or at least six episodes in a shorter period. At least two episodes of inappropriate compensatory behaviors per month for at least three months or at least six episodes in a shorter period. Undue influence of body weight and shape on self-evaluation.
Binge eating disorder (BED)	At least four episodes or days of uncontrollable binge eating per month for at least three months. Fewer than one inappropriate compensatory behavior on average per month during this period. Marked distress about binge eating. Binge eating characterized by three or more of the following: rapid eating; eating until uncomfortably full; eating large amounts of food when not hungry; eating alone due to embarrassment; feeling disgusted, depressed, or guilty after overeating.
Subthreshold BED	At least two episodes or days of uncontrollable binge eating per month for at least three months or at least six episodes in a shorter period. Fewer than one inappropriate compensatory behavior on average per month during this period. Marked distress about binge eating. Binge eating characterized by three or more of the following: rapid eating; eating until uncomfortably full; eating large amounts of food when not hungry; eating alone due to embarrassment; feeling disgusted, depressed, or guilty after overeating.
Purging disorder (PD)	At least four episodes of self-induced vomiting and/or diuretic/laxative use to control weight per month for at least three months. Fewer than one episode of uncontrollable binge eating on average per month during this period. Undue influence of body weight and shape on self-evaluation.

**Table 2:** Baseline prevalence and future onset of eating disorders across ethnic/racial groups.

Ethnicity/Race	Baseline ED Cases (%)	Future ED Cases (%)
White	28 (67)	201 (61)
Asian	6 (14)	45 (14)
Black	3 (7)	16 (5)
Hispanic	4 (10)	56 (17)
Native American	1 (2)	10 (3)

*Note.* Those who had any eating disorders at baseline (*n* = 42) were not included in the major analyses.

**Table 3:** Predictive effects of risk factors across ethnic/racial groups.

Risk Factors	Comparison Group	LOR	OR	SE	Z	*p*	Adjusted *p*	OR 95% CI		SRD
Main Effects										
Thin-ideal internalization	-	0.80	2.22	0.17	4.58	**<0.001**	**0.006**	1.58	3.13	0.23
Body dissatisfaction	-	0.67	1.95	0.11	5.83	**<0.001**	**0.006**	1.56	2.45	0.26
Negative affect	-	0.68	1.97	0.08	8.71	**<0.001**	**0.006**	1.69	2.30	0.37
Restrictive eating	-	0.86	2.37	0.10	8.67	**<0.001**	**0.006**	1.96	2.89	0.40
Psychosocial impairment	-	0.92	2.50	0.14	6.34	**<0.001**	**0.006**	1.89	3.32	0.26
zBMI	-	−0.22	0.80	0.08	−2.60	**0.** **009**	**0.** **039**	0.68	0.95	0.12
Interactions										
Thin-ideal internalization	Asian	0.50	1.65	0.46	1.09	0.276	0.753	0.69	4.15	0.23
	Black	−0.05	0.95	0.60	−0.08	0.933	0.978	0.31	3.42	0.22
	Hispanic	0.13	1.14	0.37	0.36	0.719	0.947	0.56	2.41	0.24
	Native	0.51	1.66	0.83	0.61	0.539	0.933	0.34	9.45	0.22
Body dissatisfaction	Asian	−0.21	0.81	0.27	−0.80	0.426	0.919	0.48	1.38	0.25
	Black	0.32	1.38	0.41	0.79	0.429	0.919	0.65	3.29	0.27
	Hispanic	0.18	1.20	0.25	0.73	0.464	0.928	0.75	1.98	0.30
	Native	0.10	1.11	0.55	0.19	0.849	0.978	0.40	3.60	0.27
Negative affect	Asian	−0.01	0.99	0.19	−0.06	0.956	0.978	0.69	1.44	0.37
	Black	−0.15	0.86	0.24	−0.64	0.522	0.933	0.54	1.39	0.36
	Hispanic	−0.14	0.87	0.15	−0.92	0.359	0.899	0.65	1.18	0.38
	Native	0.17	1.18	0.46	0.35	0.726	0.947	0.50	3.13	0.36
Restrictive eating	Asian	−0.04	0.96	0.25	−0.16	0.871	0.978	0.60	1.58	0.40
	Black	−0.67	0.51	0.34	−1.97	**0.049**	0.184	0.27	1.01	0.39
	Hispanic	−0.12	0.89	0.21	−0.54	0.591	0.933	0.59	1.37	0.41
	Native	0.01	1.01	0.47	0.03	0.978	0.978	0.44	2.83	0.40
Psychosocial impairment	Asian	0.07	1.07	0.40	0.18	0.856	0.978	0.50	2.38	0.27
	Black	0.05	1.05	0.50	0.11	0.915	0.978	0.41	2.95	0.27
	Hispanic	−0.43	0.65	0.32	−1.34	0.181	0.603	0.35	1.23	0.27
	Native	0.34	1.40	0.73	0.46	0.643	0.947	0.35	6.54	0.27
zBMI	Asian	0.12	1.13	0.22	0.57	0.572	0.933	0.73	1.72	0.13
	Black	0.68	1.98	0.25	2.77	**0.006**	**0.030**	1.22	3.25	0.15
	Hispanic	0.07	1.07	0.17	0.38	0.701	0.947	0.76	1.48	0.16
	Native	0.46	1.58	0.37	1.24	0.214	0.642	0.76	3.35	0.13

*Note*. Reference group = White women. Native = Native American. LOR = log odds ratio; OR = odds ratio; SRD = success rate difference. SRDs of 0.11, 0.28, and 0.43 correspond to small, medium and large effects, respectively. Significant results are in bold.

**Table 4:** Predictive effects of prodromal symptoms across ethnic/racial groups.

Risk Factors	Comparison Group	LOR	OR	SE	Z	*p*	Adjusted *p*	OR 95% CI		SRD	
Main Effects											
Binge eating	-	0.25	1.28	0.03	7.57	**<0.001**	**0.005**	1.20	1.37	0.37	
Compensatory behavior	-	0.34	1.40	0.05	6.99	**<0.001**	**0.005**	1.27	1.53	0.37	
Wt./shape overvaluation	-	0.54	1.72	0.07	7.86	**<0.001**	**0.005**	1.50	1.97	0.39	
Fear of weight gain	-	0.28	1.32	0.03	8.41	**<0.001**	**0.005**	1.23	1.40	0.34	
Feeling fat	-	0.25	1.28	0.04	6.80	**<0.001**	**0.005**	1.19	1.38	0.30	
Interactions											
Binge eating	Asian	0.14	1.15	0.10	1.37	0.172	0.538	0.95	1.41	0.37	
	Black	0.67	1.95	0.28	2.38	**0.017**	0.071	1.18	3.67	0.36	
	Hispanic	0.13	1.14	0.09	1.45	0.148	0.529	0.96	1.38	0.37	
	Native	0.09	1.09	0.17	0.51	0.614	0.988	0.79	1.60	0.36	
Compensatory behavior	Asian	−0.01	0.99	0.13	−0.06	0.952	0.988	0.76	1.30	0.35	
	Black	0.07	1.07	0.17	0.43	0.666	0.988	0.78	1.53	0.38	
	Hispanic	−0.04	0.96	0.10	−0.40	0.689	0.988	0.79	1.18	0.36	
	Native	0.10	1.10	0.21	0.43	0.666	0.988	0.78	1.81	0.37	
Wt./shape overvaluation	Asian	0.10	1.10	0.18	0.55	0.582	0.988	0.78	1.60	0.39	
	Black	−0.12	0.89	0.23	−0.53	0.598	0.988	0.58	1.43	0.37	
	Hispanic	0	1.00	0.16	−0.02	0.988	0.988	0.73	1.39	0.38	
	Native	0.07	1.07	0.34	0.21	0.832	0.988	0.58	2.20	0.38	
Fear of weight gain	Asian	−0.05	0.95	0.08	−0.57	0.570	0.988	0.81	1.12	0.35	
	Black	0.04	1.04	0.13	0.31	0.755	0.988	0.81	1.36	0.35	
	Hispanic	−0.01	0.99	0.08	−0.17	0.865	0.988	0.85	1.15	0.36	
	Native	0.02	1.02	0.15	0.16	0.870	0.988	0.77	1.40	0.35	
Feeling fat	Asian	0.04	1.04	0.09	0.42	0.675	0.988	0.87	1.24	0.32	
	Black	−0.13	0.88	0.13	−1.03	0.306	0.850	0.68	1.13	0.29	
	Hispanic	0	1.00	0.08	0.06	0.955	0.988	0.85	1.19	0.32	
	Native	0.06	1.06	0.21	0.28	0.779	0.988	0.73	1.72	0.30	

*Note*. Reference group = White women. Native = Native American. Wt./shape overvaluation = weight/shape overevaluation; LOR = log odds ratio; OR = odds ratio; SRD = success rate difference. SRDs of 0.11, 0.28, and 0.43 correspond to small, medium and large effects, respectively. Significant results are in bold.

## Data Availability

The raw data supporting the conclusions of this article will be made available by the authors on request.

## Abbreviations

The following abbreviations are used in this manuscript:

AN	Anorexia nervosa
BN	Bulimia nervosa
BED	Binge eating disorder
PD	Purging disorder
OSFED	Other specified feeding and eating disorder
